# The role of job satisfaction on the association between perceived person-centred care and stress of conscience: a moderated mediation analysis of cross-sectional data from primary care professionals

**DOI:** 10.1186/s12875-025-03163-8

**Published:** 2026-01-15

**Authors:** Cornelia van Diepen, Kristoffer Gustavsson, Gunnel Hensing, Qarin Lood, Andreas Fors

**Affiliations:** 1 Erasmus School of Health Policy & Management, Erasmus University Rotterdam, Rotterdam, Netherlands; 2 Gothenburg Centre for Person-Centred Care (GPCC), University of Gothenburg, Gothenburg, Sweden; 3 Institute of Health and Care Sciences, Sahlgrenska Academy, University of Gothenburg, Goteborg, Sweden; 4 School of Public Health and Community Medicine, Institute of Medicine, Sahlgrenska Academy, University of Gothenburg, Gothenburg, Sweden; 5 Institute of Neuroscience and Physiology, Department of Health and Rehabilitation, Sahlgrenska Academy, University of Gothenburg, Gothenburg, Sweden; 6 Region Västra Götaland, Research, Education, Development and Innovation, Primary Health Care, Gothenburg, Sweden

**Keywords:** Primary care, Person-centred care, Stress of conscience, Job satisfaction, Healthcare professionals

## Abstract

**Background:**

Primary care professionals play a crucial role in promoting public health, yet they often face challenging work environments characterised by stress of conscience, described as distress felt when external constraints or conflicting responsibilities prevent healthcare professionals from acting in line with their ethical beliefs. Introducing more person-centred care (PCC) could increase job satisfaction and mitigate these challenges. This study aimed to determine the association between perceived PCC and the experience of stress of conscience among primary care professionals and whether this association is mediated by job satisfaction and moderated by profession.

**Methods:**

A cross-sectional survey methodology was employed, involving various primary care professionals in western Sweden. Of the 944 completed questionnaires (21.3% response rate), 562 were included in the analysis based on specific criteria. The questionnaire measured their perceived PCC, job satisfaction, type of profession, and stress of conscience. Statistical analyses, including mediation analysis and moderated mediation analysis, were performed.

**Results:**

The findings show that perception of more PCC was significantly associated with lower levels of stress of conscience. Furthermore, mediation analysis revealed that job satisfaction partially mediated the relationship between perceived PCC and stress of conscience. Our study found that approximately 29% of the total effect of perceived PCC on stress of conscience was transmitted through job satisfaction. Including the type of profession as a moderator led to a decrease in the mediated association between perceived PCC and stress of conscience (*B* = -1.443 to *B* = -0.786).

**Conclusions:**

This study showed that perceived PCC was directly associated with stress of conscience in primary care settings, with job satisfaction partially mediating this association. Future studies should consider job satisfaction as a mediating factor and explore the impact of professional variation on the PCC adoption and effectiveness.

**Supplementary Information:**

The online version contains supplementary material available at 10.1186/s12875-025-03163-8.

## Background

Healthcare professionals in primary care are key to reaching equal and good public health [1, 2]. However, their work environment is often challenging, marked by high demands, low control, demanding work schedules, heavy workloads [3, 4], and stress of conscience [5, 6]. Previous research e.g., [7–9] has highlighted that job dissatisfaction, poor working conditions, and work-related stress are factors driving turnover and sickness absence among healthcare professionals. A recent cross-sectional study involving almost 3,000 physicians in Swedish primary care found that nearly one-fourth reported dissatisfaction with their jobs [10]. Therefore, the work environment in primary care is an important starting point for promoting job satisfaction and reducing stress of conscience.

Person-centred care (PCC) is a care model grounded in ethical principles that recognises, and is guided by, patients’ resources and needs, identified through listening to the patient’s narrative. Decision-making and care-planning are conducted collaboratively, integrating the patient’s perspective with the expertise of healthcare professionals and promoting teamwork across healthcare professionals [11]. Several PCC interventions in different healthcare settings, including primary care, have been shown to improve patients’ self-efficacy, symptom control, and satisfaction with care, while also reducing length of hospital stay and healthcare costs [12, 13]. Higher perceived PCC is also associated with increased job satisfaction (Van Den Pol-Grevelink et al., 2012; van Diepen et al., 2020), job productivity, and organisational commitment [14], as well as reduced burnout [15], and stress of conscience (van Diepen et al., 2021) among healthcare professionals.

Stress of conscience can be defined as the emotional and psychological distress experienced by people, particularly healthcare professionals, when they feel unable to act according to their moral or ethical beliefs due to external constraints or conflicting responsibilities [16]. Prolonged work intensification, personal worries, and lack of social support have been linked to higher levels of stress of conscience [6, 17, 18]. Stress of conscience has also shown associations with declined quality of patient care [5], and reduced job satisfaction among healthcare professionals [19]. Orrung Wallin et al., [19] points out that although job satisfaction and stress of conscience overlap conceptually to a certain degree, the results from their study show that they can be differentiated. Stress of conscience has also shown connections to burnout [5], which in turn is related to increasing turnover rates and difficulties of retaining staff in healthcare [20]. Longitudinal research by Herttalampi & Feldt [21] showed that situations which might trouble the conscience, such as having to provide care of lower quality due to time constraints, was a statistically significant risk factor for burnout and turnover intentions.

Job satisfaction among healthcare professionals is crucial for increased productivity and commitment to the organisation [22]. A recent study with Swedish midwives found a strong association between job satisfaction and factors such as opportunities for development, quality of work, role conflict, burnout, and recognition [23]. These elements overlap and connect with stress of conscience within healthcare and social services [5, 24]. Therefore, we propose that job satisfaction could mediate the relationship between perceived PCC and stress of conscience.

The association between perceived PCC and stress of conscience may vary among professional groups due to differing responsibilities and tasks. For instance, Munkeby et al. [25] found that registered nurses experienced more stress of conscience than other healthcare professionals in long-term care. Similarly, Gustavsson et al. [26] reported that physicians had the highest mean stress of conscience score and the lowest perception of PCC in a Swedish study with hospital and municipality staff. In contrast, assistant nurses experienced higher PCC and lower stress of conscience [26]. Additionally, factors associated with job satisfaction can differ between nurses and physicians, as shown in a multi-centre cross-sectional study from several European countries [9].

**Fig. 1:**
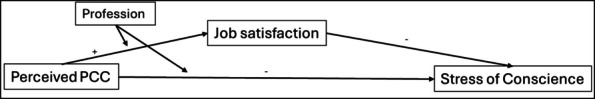
Expected mediated relationship among perceived person-centred care (PCC), job satisfaction, and stress of consciences as well as the moderating effects of type of profession

Based on the scientific literature, we present the following model in Fig. 1. We analysed the association between perceived PCC and stress of conscience, considering the potential mediation of job satisfaction. Additionally, we tested whether these associations were moderated by profession.

## Aim

The study aimed to determine the association between perceived PCC and the experience of stress of conscience among healthcare professionals in primary care, and whether this association was mediated by job satisfaction and moderated by profession.

## Methods

### Study design

This was a cross-sectional survey study with healthcare professionals working in Swedish primary care. The study is part of the prospective, longitudinal PCC@Work project on work-related health among health and social care professionals across multiple care settings, described in detail elsewhere [27], and applies the baseline data from the participating primary care organisations. Analyses of municipal and hospital care baseline data can be found in Gustavsson et al. [26].

The study has been approved by the Swedish Ethical Review Authority (DNr: 2019–01287, 2021–02821, 2021–05094 and 2023–05980–02), and it is reported in line with the Strengthening the Reporting of Observational Studies in Epidemiology (STROBE) checklist for cross-sectional studies (Additional file 1) [28].

### Setting and participants

In Sweden, primary care is responsible for providing medical assessments, treatment, preventive care, and rehabilitation that do not require specialised medical or technical resources (Swedish Health and Medical Services Act [Hälso- och sjukvårdslagen 2017:30]).

Responsibility is divided between regions (*n* = 21) and municipalities (*n* = 290). Generally, primary care is funded by regional taxes and patient fees, with geographical variations concerning how primary care services are organised and delivered [29]. This study was conducted across 105 regional primary care centres, midwifery and gynaecology clinics, as well as privately operated rehabilitation clinics in western Sweden. The last author contacted unit managers of all involved organisations to obtain the work email addresses of employed healthcare professionals.

### Data collection

Data were collected using a web-based survey developed by the research team and distributed by an experienced data collection company. An email, including information about the study and an individual link to the survey, was sent to 4422 healthcare professionals in primary care between May and June 2024. A total of 944 healthcare professionals responded, resulting in a 21.3% response rate. The survey, conducted in Swedish, included questions on sociodemographic data, perceived PCC, stress of conscience, and job satisfaction. Three reminder emails were sent during the data collection period. The data collection company organised the database for analysis but played no part in the analysis.

### Measures

#### Perceived Person-Centred Care (PCC)

The exposure variable was the degree of perceived PCC in the workplace measured using an adapted version of the Person-Centred Care Assessment Tool (P-CAT) described in Gustavsson et al. [26]. The adapted P-CAT was designed for use in various health and social care settings. Similar to the original validated tool developed by Edvardsson et al., [30], the adjusted version contains 13 items. In each item, professionals rate how they perceive the care on a five-point scale ranging from 1 (‘disagree completely’) to 5 (‘agree completely’). A total score between 13–65 is calculated, with a higher score signifying a higher degree of perceived PCC. The current dataset generated satisfactory psychometric quality based on a factor analysis with Kaiser–Meyer–Olkin’s measure of sampling adequacy of 0.85 (*p* < 0.001) and a two-factor solution explaining 52.3% of the variance. It also showed satisfactory reliability estimates with a total Cronbach’s α of 0.84.

#### Stress of conscience

The outcome variable, stress of conscience, was measured using the Stress of Conscience Questionnaire (SCQ) [16]. The questionnaire consists of nine A-statements and nine B-statements that assess how frequently stressful situations occur and the extent to which they trouble the participant’s conscience. The A-statements assess the frequency of stressful situations on a scale from 0 (‘Never’) to 5 (‘Every day’), while B-statements assess the degree to which these situations trouble the participant’s conscience, ranging from 0 (‘No, not at all’) to 5 (‘Yes, a very troubled conscience’). In the web-based survey, B-statements were omitted for participants who responded 0 (‘Never’) to the corresponding A-statement. Each A-statement score is multiplied by its corresponding B-statement score, resulting in a total score ranging from 0 to 225 [16, 31], with higher scores indicating greater stress of conscience.

The psychometric properties of the SCQ have been shown to be satisfactory and reliable for use in the Swedish health and social care context [16, 17]. Kaiser–Meyer–Olkin for the SCQ in this dataset was 0.89 (*p* < 0.001), with one factor explaining 59.9% of the variance. The Cronbach’s α for the total SCQ score in this dataset was 0.86.

#### Job satisfaction

Job satisfaction was measured using four items from the Copenhagen Psychosocial Questionnaire (COPSOQ III) [32]. The prompt was: “Regarding your work in general, how pleased are you with…” followed by: your work prospects, the physical working conditions, the way your abilities are used, and your job as a whole, taking everything into consideration. Response options were: Very satisfied (100), Satisfied (75), Neither/Nor (50), Unsatisfied (25), and Very unsatisfied (0). The responses to the four questions were averaged to generate a score between 0 and 100, with higher scores indicating greater job satisfaction. The items were combined to reflect overall job satisfaction, as not all can be independently influenced by perceived PCC.

The job satisfaction variable has been satisfactorily validated in a Swedish context [32]. In this study, Kaiser–Meyer–Olkin for job satisfaction was 0.80 (*p* < 0.001), with one factor explaining 68.2% of the variance, and Cronbach’s α was 0.84.

#### Type of profession

The question about the participants’ type of profession included 13 predefined options, along with an open-ended category for those whose profession was not listed. A categorisation of the answers yielded six categories of professions: physician, psychologist (also including counsellor), registered nurse (RN), assistant nurse (AN), midwife, and ‘other’, which for example included occupational therapist, physiotherapist, and podiatrist.

The ‘other’ category was used as the control variable in the analyses to provide a broad baseline of varying professions. This allowed for comparisons between the larger single-profession groups and a more diverse reference category, highlighting the differences between specific professional groups and the broader baseline.

#### Other variables

The survey also included demographic and work-related variables that could be considered confounders, of which this study included gender, age, previous experience with PCC, and years of work experience in healthcare. However, when they showed no significant connection to the stress of conscience or sufficient impact in the analysis, they were excluded from further analyses.

### Statistical analyses

IBM SPSS Statistics version 29 (IBM [33]) and the PROCESS macro for SPSS version 4.2 [34] were used to perform the analyses. P-values below 0.05 were considered statistically significant. This study applied an adequate sample size. A power analysis was conducted using the G*Power software version 3.1.9.7 [35] to determine the required sample size for the moderated mediation analysis. To detect a small effect (f2 = 0.02) with a power of 0.80 (α = 0.05), a minimum sample size of 518 was required.

Responses with less than 50% missing items in P-CAT (maximum six missing) and SCQ (maximum four missing) were imputed with the total scale mean [36]. No systematic missing values were identified.

The four negatively worded items in the version of the P-CAT used in this study (items 8, 9, 10, and 11) were reversed before analysis. Descriptive statistics are presented in stratified groups, showing their quantity and percentage within the variable. Moreover, the one-way ANOVA provided the mean and standard deviation of the SCQ scores and indicated if there were statistically significant differences per group. Bivariate associations between the study variables were analysed using Spearman correlation analysis, as the stress of conscience variable was skewed.

Assumptions for moderated mediation analysis were tested. Variance inflation factors were under 10 (1.06–1.69), indicating no multicollinearity [37]. Visualisation of scatter plots showed no outliers, approximate linearity, and mild heteroscedasticity. An inspection of the histogram and Probability-Probability plot showed only minor deviations from the normal distribution in residual errors [38]. Since mild heteroscedasticity is not considered a concern, and linear regressions are robust against minor deviations in normality [34], we proceeded with a moderated mediation analysis [38].

Subsequently, a moderated mediation analysis was executed with perceived PCC as the independent variable, job satisfaction as the mediator, stress of conscience as the dependent variable, and type of profession as the moderator (see Fig. 1). Moderated mediation implies that the moderator alters the strength or direction of the indirect effect of the independent variable on the dependent variable via the mediator [34]. The association between the variables were calculated using Hayes’ PROCESS models 4 and 8 [34]. The moderated mediation analysis contained the following steps:

1. The mediation analysis (PROCESS Model 4) was performed to test whether job satisfaction mediated the relationship between perceived PCC and stress of conscience, with and without type of profession and age as covariates.
2. A multiple regression analysis was conducted to estimate the effects of perceived PCC and the moderator ‘type of profession’ on job satisfaction and the interaction effect between perceived PCC and the moderator ‘type of profession’ (PROCESS Model 8).
3. A multiple regression analysis was conducted with stress of conscience as the dependent variable to estimate the effects of perceived PCC, job satisfaction, and type of profession and the interaction effect between perceived PCC and the moderator (PROCESS model 8).

## Results

Out of the 944 participants, 562 completed more than 50% of the P-CAT and the SCQ items and, therefore, were included in the study. The majority worked in publicly funded primary care centres (*n* = 452, 80.4%), while the remaining participants were employed in midwifery and gynaecology clinics or private rehabilitation clinics (*n* = 110, 19.6%). 

### Descriptive statistics

Table 1 shows that most participants were women (*n* = 488, 86,8%). The mean stress of conscience score between age groups revealed a statistically significant difference, with older participants reporting higher scores than younger ones. Almost three-quarters of participants (*n* = 412, 73.8%) had more than 10 years of experience working in healthcare and reported significantly lower mean stress of conscience scores than the other groups. Most participants (*n* = 449, 79,9%) had previous experience working with PCC.

**Table 1:** Demographic characteristics of participants and mean stress of conscience levels, Swedish primary care professionals (*n* = 562), PCC@Work, 2024 [27]

	**Total sample**	
**Variables**	***n* (%)**^**a**^	**Stress of conscience mean ± SD**
	562	46.0 ± 39.1
Gender	562	
Women	488 (86.8)	47.1 ± 39.4 *ns*
Men	70 (12.5)	39.7 ± 37.5
Other	4 (0.7)	40.3 ± 10.1
Age	535	
≤ 30	205 (38.3)	38.9 ± 35.4 **
31–40	151 (28.2)	44.52 ± 37.7
41–50	140 (26.2)	55.7 ± 43.0
> 51	39 (7.3)	59.3 ± 39.0
Perceived person-centred care score^b^	562	
≤ 25	9 (1.6)	97.8 ± 47.6**
26–38	108 (19.2)	66.9 ± 44.9
39–51	286 (50.9)	48.1 ± 37.2
> 52	159 (28.3)	25.2 ± 23.7
Job satisfaction^b^	551	
Very unsatisfied (≤ 25)	10 (1.8)	70.6 ± 62.9**
Unsatisfied (26–50)	49 (8.9)	79.7 ± 40.1
Satisfied (51- 75)	148 (33.4)	58.5 ± 40.6
Very satisfied (≥ 76)	308 (55.9)	32.2 ± 29.8
Type of profession	560	
Physician	86 (15.3)	57.4 ± 39.4 *ns*
Psychologist (including counsellor)	73 (13.0)	48.3 ± 39.7
Registered nurse (including specialist nurse)	176 (31.3)	46.7 ± 36.9
Assistant nurse	63 (11.2)	42.9 ± 39.8
Midwife	69 (12.3)	50.4 ± 39.8
Other professions (e.g., occupational therapist, physiotherapist, podiatrist)	93 (16.5)	32.0 ± 38.2
Years of work experience healthcare	558	
< 3 years	16 (2.9)	46.3 ± 25.6*
3–5 years	50 (9.0)	± 43.1
6–10 years	80 (14.3)	56.6 ± 46.5
> 10 years	412 (73.8)	43.9 ± 37.2
Previous experience working with person-centred care	562	
Yes	449 (79.9)	48.3 ± 39.6 *ns*
No	62 (11.0)	43.3 ± 40.2
Not sure	51 (9.1)	29.5 ± 27.8

*SD* Standard deviation

^*^ns = not significant; *p* < 0.05; **p < 0.001 (two-tailed) in Chi-square test

^a^Numbers do not always add up to 562 due to internal attrition

^b^These variables are categorised but will be analysed as continuous due to undefined cut-off points

### Bivariate associations

Perceived PCC was negatively correlated with stress of conscience (−0.429**) and positively correlated with job satisfaction (0.461**), indicating that participants who reported higher levels of perceived PCC experienced lower stress of conscience and greater job satisfaction (Table 2). Additionally, higher stress of stress of conscience scores correlated with lower job satisfaction (−0.452**).

**Table 2:** Bivariate correlations of the main study variables (*n* = 562)

Correlations			
	1. Perceived person-centred care	2. Stress of conscience	3. Job satisfaction
1. Perceived person-centred care			
2. Stress of conscience	−0,429**		
3. Job satisfaction	0,461**	−0,452**	
4a. Physician	−0,036	0,133**	−0,005
4b. Psychologist	0,067	0,025	0.29
4c. Registered nurse	−0,054	0,039	0,072
4d. Assistant nurse	−0,046	−0,037	0,035
4e. Midwife	0,060	0,048	−0,050
4f. Other	0,026	−0,208**	−0,097*

^*^p < 0.05; **p < 0.001 (two-tailed)

Although most professions were not significantly correlated with the main study variables, Table 2 shows variations in the direction of the correlations. Notably, being a physician significantly correlated with higher stress of conscience, while the ‘other’ category had significantly lower stress of conscience than the other professions.

### Mediation results

Higher levels of perceived PCC were positively associated with greater job satisfaction (*B* = 1.017**;* R*^*2*^ = 0.220) (Table 3). The negative association in the second row (*B* = −0.573**; *R*^*2*^ = 0.277) indicates that greater job satisfaction is associated with lower stress of conscience. The total effect of perceived PCC on stress of conscience was negative, with path coefficients from −2.025** to −1.966**, suggesting that higher perceived PCC was linked to lower stress of conscience, mediated by job satisfaction. The direct effect of perceived PCC on stress of conscience remained negative (from *B* = −1.443** to* b* = −1.400**) even after controlling for profession and age, suggesting an association between perceived PCC and stress of conscience which is partially mediated by job satisfaction. Specifically, 28.78% of the total effect was mediated by job satisfaction.

**Table 3:** The mediated model of the results adjusted for profession and age (*n* = 551)

	**Model 1**			**Model 2 (with type of profession)**			**Model 3 (with type of profession and age)**		
	*B*	*SE*	*R*^*2*^	*B*	*SE*	*R*^*2*^	*B*	*SE*	*R*^*2*^
*Perceived PCC – Job satisfaction*	1.017**	0.082	0.220	1.015**	0.082	0.223	0.998**	0.082	0.240
*Job satisfaction – Stress of conscience*	−0.573**	0.083	0.277	−0.584**	0.083	0.283	−0.565**	0.085	0.330
*(Total) Perceived PCC – Stress of conscience*	−2.025**	0.165	0.215	−2.023**	0.165	0.218	−1.966**	0.165	0.271
*(Direct) Perceived PCC – Stress of conscience*	−1.443**	0.180	0.215	−1.430**	0.180	0.218	−1.400**	0.180	0.271

*SE* Standard error

^*^p < 0.05; **p < 0.001 (two-tailed)

R^2^ = Adjusted R-squared

*B* = Unstandardised beta coefficient

### Moderated mediation results

The multiple regression analysis with type of profession as the moderator revealed significant results in the B-coefficients for perceived PCC and Job satisfaction (Table 4). There were minor changes in the B-coefficient (from B = 1.017** to 0.979**) between perceived PCC and job satisfaction, indicating a weakening association when perceived PCC interacted with the participants' type of profession.

**Table 4:** Results of the multiple regression analyses as part of the moderated mediation analyses with job satisfaction and stress of conscience as dependent variables (*n* = 549)

Predictors	Dependent variable: Job satisfaction		Dependent variable: Stress of conscience	
	*B*	*B*	*B*	*B*
Perceived person-centred care (PCC)	1.017**	0.979**	−1.443**	−0.786*
Job satisfaction			−0.573**	−0.620**
Profession (‘Other’ is the reference)				
4a. Physician		−1.310		39.813
4b. Psychologist		−9.414		76.440
4c. Registered nurse (RN)		10.278		50.136
4d. Assistant nurse (AN)		13.937		53.679
4e. Midwife		−10.313		66.221
Perceived PCCx profession				
Perceived PCCx4a. Physician		0.166		−0.267
Perceived PCCx 4b. Psychologist		0.303		−1.150*
Perceived PCCx 4c. RN		−0.045		−0.672
Perceived PCCx 4d. AN		−0.127		−0.900
Perceived PCCx 4e. Midwife		0.245		−0.959
	*R*^*2*^ = *0.215 F(1, 549)* = 154.5**	*R*^*2*^ = *0.251 F(11, 537)* = 16.39**	*R*^*2*^ = *0.277 F(2, 548)* = 105.33**	*R*^*2*^ = *0.333 F(12, 536)* = 22.30**

^*^p < 0.05; **p < 0.001 (two-tailed)

*R*^2^ = Adjusted R-squared

*b* = Unstandardised beta coefficient

The second and central part of the model showed substantial differences between perceived PCC and stress of conscience with job satisfaction as a mediator, with the B-coefficient decreasing from −1.443** to −0.786* with the inclusion of the moderator, although no significant moderated mediation was present. The interaction terms show the direction of potential differences in how perceived PCC’s impact varies by type of profession. A positive interaction term suggests that perceived PCC boosts job satisfaction more for physicians, psychologists, and midwives than the ‘others’, but none of these reached statistical significance. Regarding stress of conscience, point estimates suggest a stronger negative association for all professions compared to the reference category, but only the interaction for psychologists was statistically significant. The interplay between perceived PCC and type of profession contributes to defining stress of conscience levels through the direct path, while the indirect path via job satisfaction was not moderated.

## Discussion

This study aimed to determine the association between perceived PCC and the experience of stress of conscience among Swedish primary care professionals, and whether this association was mediated by job satisfaction and moderated by profession. The type of profession did not significantly moderate the mediation of PCC, stress of conscience and job satisfaction, but did influence the results in the model, suggesting that profession may be an important factor to consider PCC in the workplace to reduce stress of conscience and enhance job satisfaction.

The results showed that higher perceptions of PCC were associated with lower stress of conscience, consistent with findings from a previous study by our group conducted in municipal and hospital care [26]. Moreover, when job satisfaction was added as a mediator, it influenced the model by demonstrating how perceived PCC combined with job satisfaction contributes to explaining lower stress of conscience. Based on the consistent patterns observed in our studies, we anticipate that similar results will be evident across other healthcare contexts.

The potential of job satisfaction as a mediator is supported by earlier cross-sectional research, which identified a connection between perceived PCC and job satisfaction [14], and by Orrung-Wallin et al. [19] , who found a negative correlation between job satisfaction and stress of conscience. Moreover, a qualitative review by our group [39] found that professionals reported increased feelings of job satisfaction after experiencing improvements in the work environment, particularly in communication and collaboration, as a result from PCC. These aspects may represent key drivers in the mediation process, reducing stress of conscience through care that is both satisfactory and ethically sound.

Previous work has shown that job satisfaction may act as a protective factor against work-related stressors that negatively affect mental health [40]. In relation to stress of conscience, it is also plausible that healthcare professionals who are less satisfied with their jobs may be more sensitive to situations that trouble their conscience. Supporting this interpretation, Dziurka et al. [41] found that registered nurses and midwives who perceived a higher degree of moral sensitivity also reported higher stress of conscience. However, the directionality of these associations remains uncertain. Future longitudinal studies are therefore needed to further investigate these pathways.

As shown in the results, physicians and midwives reported higher stress of conscience compared to other professional groups, consistent with previous studies e.g., [5, 42]. These profession-specific differences in the perception of PCC should be carefully considered. For instance, a review on PCC in primary care [43] highlights the importance of involving the entire healthcare workforce in discussions about PCC to better understand how to achieve it. Similarly, a meta-synthesis on PCC implementation [44] found that different professions hold varying views and expectations of PCC and apply the approach in different ways. This aligns with findings in the above-mentioned qualitative review by Gustavsson et al. [39], which identified how healthcare professionals experienced a shift in their professional role when applying PCC, sometimes experienced as challenging or uncertain due to new ways of working [39]. Healthcare professionals thus need appropriate tools to facilitate PCC Grover et al. [45], and our findings suggest a need for primary care and other healthcare organisations to target PCC implementation efforts and evaluate PCC in relation to the goal of reducing stress of conscience across professional groups, either directly or through improved job satisfaction.

### Limitations

Certain study limitations must be considered in the present study. Due to its cross-sectional design, the direction of causality could not be determined, and the direction of the mediation could be reversed. The response rate was 21.3% among all the healthcare professionals contacted. Although the number of responses was anticipated, and a power calculation confirmed that the sample size was sufficient for our study, the possibility of selection bias remains. In the present model, several concepts were considered as potential confounders, but age, gender, and years of experience were not significantly related to more than one concept. This indicates that, while these factors remain relevant for understanding PCC in the primary care context, they were not the primary drivers of the relationships identified in this study.

Moreover, it is possible that there is a common-method bias as primary care professionals who are content with their jobs or interested in PCC might have been more likely to respond to the web-based survey, which could imply an overestimation of PCC levels. In addition, since PCC is being integrated throughout Swedish healthcare, participants may have overestimated the extent of PCC at their workplaces. Lastly, the job satisfaction score was based on four items that together constituted the study's variable. The individual items, however, could not serve as mediating factors on their own.

## Conclusion

The findings of this study indicate that job satisfaction may serve as a mediating factor in the association between perceived PCC and stress of conscience. Despite initial expectations, the moderated mediation analysis showed no significant professional differences. Nevertheless, recognising that healthcare professionals may still have distinct experiences and perceived needs remains important for the broader implementation of PCC. Understanding these perspectives can help healthcare organisations develop strategies that address common challenges while also accommodating contextual differences in daily practice. Ultimately, such efforts may contribute to improving the work-related health of healthcare professionals and enhancing the quality of patient care. These insights underscore the need for further longitudinal research and implementation strategies that incorporate the role of job satisfaction while also exploring, rather than assuming, the influence of professional variations in the adoption and effectiveness of PCC.

## Electronic Supplementary Material

Below is the link to the electronic supplementary material.

Additional file 1

## Data Availability

The datasets generated and analysed as part of this study are not publicly available. Data used and/or analysed in this study can be provided in de-identified form by the corresponding author upon reasonable request. All data are covered by the Swedish Public Access to Information and Secrecy Act (Offentlighets- och sekretesslagen), and a confidentiality assessment (sekretessprövning) will be performed for each individual request. Permission from the Institute of Health and Care Sciences, University of Gothenburg, must be obtained before data can be accessed. Data will be stored for 10 years after publication at the University of Gothenburg, Sweden.

## Abbreviations

WHO: World Health Organization
PCC: Person-centred care
P-CAT: Person-Centred Care Assessment Tool
SCQ: Stress of Conscience Questionnaire
STROBE: Strengthening the Reporting of Observational Studies in Epidemiology
COPSOQ III: Copenhagen Psychosocial Questionnaire
KMO: Kaiser-Meyer-Olkin

## References

[1] WHO. Operational Framework for Primary Health Care: Transforming Vision Into Action (Technical Series on Primary Health Care). World Health Organization and the United Nations Children’s Fund (UNICEF). ISBN (WHO) 978–92–4–001783–2 (electronic version). 2020.

[2] WHO - Primary health care. WHO - Primary Health Care. n.d. Retrieved 21 January 2025, from https://www.who.int/health-topics/primary-health-care.

[3] Anskär E, Lindberg M, Falk M, Andersson A. Time utilization and perceived psychosocial work environment among staff in Swedish primary care settings | SpringerLink. BMC Health Serv Res. 2018;18(166). 10.1186/s12913-018-2948-6. 10.1186/s12913-018-2948-6 PMC5842529 29514637

[4] Halter M, Boiko O, Pelone F, Beighton C, Harris R, Gale J, et al. The determinants and consequences of adult nursing staff turnover: a systematic review of systematic reviews. BMC Health Serv Res. 2017;17(1):824. 10.1186/s12913-017-2707-0. 29246221 10.1186/s12913-017-2707-0 PMC5732502

[5] Jokwiro Y, Wilson E, Bish M. The extent and nature of stress of conscience among healthcare workers: a scoping review. Appl Nurs Res. 2022;63:151554. 10.1016/j.apnr.2021.151554. 35034704 10.1016/j.apnr.2021.151554

[6] Taipale M, Herttalampi M, Muotka J, Mauno S, Feldt T. Stress of conscience in healthcare in turbulent times: a longitudinal study. Nurs Ethics. 2024;31(5):805–17. 10.1177/09697330231204949. 37888885 10.1177/09697330231204949 PMC11370163

[7] Helgesson M, Marklund S, Gustafsson K, Aronsson G, Leineweber C. Interaction effects of physical and psychosocial working conditions on risk for sickness absence: a prospective study of nurses and care assistants in Sweden. Int J Environ Res Public Health. 2020;17:7427. 10.3390/ijerph17207427. 33053900 10.3390/ijerph17207427 PMC7601317

[8] Koch P, Zilezinski M, Schulte K, Strametz R, Nienhaus A, Raspe M. How perceived quality of care and job satisfaction are associated with intention to leave the profession in young nurses and physicians. Int J Environ Res Public Health. 2020;17(8):8. 10.3390/ijerph17082714. 10.3390/ijerph17082714 PMC7216191 32326518

[9] Maniscalco L, Enea M, de Vries N, Mazzucco W, Boone A, Lavreysen O, et al. Intention to leave, depersonalisation and job satisfaction in physicians and nurses: a cross-sectional study in Europe. Sci Rep. 2024;14(1):2312. 10.1038/s41598-024-52887-7. 38282043 10.1038/s41598-024-52887-7 PMC10822871

[10] Fernemark H, Karlsson N, Skagerström J, Seing I, Karlsson E, Brulin E, et al. Psychosocial work environment in Swedish primary healthcare: a cross-sectional survey of physicians’ job satisfaction, turnover intention, social support, leadership climate and change fatigue. Hum Resour Health. 2024;22(1):70. 10.1186/s12960-024-00955-4. 39443998 10.1186/s12960-024-00955-4 PMC11500482

[11] Ekman I, Swedberg K, Taft C, Lindseth A, Norberg A, Brink E, et al. Person-centered care—ready for prime time. Eur J Cardiovasc Nurs. 2011;10(4):248–51. 10.1016/j.ejcnurse.2011.06.008. 21764386 10.1016/j.ejcnurse.2011.06.008

[12] Britten N, Ekman I, Naldemirci Ö, Javinger M, Hedman H, Wolf A. Learning from Gothenburg model of person centred healthcare. BMJ (Clinical Research Ed.). 2020;370. 10.1136/bmj.m2738. 10.1136/bmj.m2738 32873594

[13] Gyllensten H, Cederberg M, Alsén S, Blanck E, Ali L, Fors A, et al. Person-centred care: state-of-the-art and future perspectives. Curr Heart Fail Rep. 2025;22(1):15. 10.1007/s11897-025-00702-3. 40214949 10.1007/s11897-025-00702-3 PMC11991961

[14] Huang CY, Weng RH, Wu TC, Hsu CT, Hung CH, Tsai YC. The impact of person-centred care on job productivity, job satisfaction and organisational commitment among employees in long-term care facilities. J Clin Nurs. 2020;29(15–16):2967–78. 10.1111/jocn.15342. 32453484 10.1111/jocn.15342

[15] Scheepers RA, Vollmann M. Nurses who co-create care with clients experience lower levels of burnout through the perception of fewer emotional demands: an observational study. BMC Nurs. 2024;23(1):902. 10.1186/s12912-024-02481-z. 39696339 10.1186/s12912-024-02481-z PMC11657821

[16] Glasberg A-L, Eriksson S, Dahlqvist V, Lindahl E, Strandberg G, Söderberg A, et al. Development and initial validation of the stress of conscience questionnaire. Nurs Ethics. 2006;13(6):633–48. 10.1177/0969733006069698. 17193804 10.1177/0969733006069698

[17] Åhlin J, Ericson-Lidman E, Eriksson S, Norberg A, Strandberg G. Longitudinal relationships between stress of conscience and concepts of importance. Nurs Ethics. 2013;20(8):927–42. 23702896 10.1177/0969733013484487

[18] Heikkilä M, Huhtala M, Mauno S, Feldt T. Intensified job demands, stress of conscience and nurses’ experiences during organizational change. Nurs Ethics. 2022;29(1):217–30. 10.1177/09697330211006831. 34374305 10.1177/09697330211006831 PMC8866741

[19] Orrung Wallin A, Edberg A-K, Beck I, Jakobsson U. Psychometric properties concerning four instruments measuring job satisfaction, strain, and stress of conscience in a residential care context. Arch Gerontol Geriatr. 2013;57(2):162–71. 10.1016/j.archger.2013.04.001. 23643346 10.1016/j.archger.2013.04.001

[20] De Vries N, Lavreysen O, Boone A, Bouman J, Szemik S, Baranski K, et al. Retaining healthcare workers: a systematic review of strategies for sustaining power in the workplace. Healthcare. 2023;11(13):1887. 10.3390/healthcare11131887. 37444721 10.3390/healthcare11131887 PMC10341299

[21] Herttalampi M, Feldt T. A new approach to stress of conscience’s dimensionality: hindrance and violation stressors and their role in experiencing burnout and turnover intentions in healthcare. J Clin Nurs. 2023;32(19–20):7284–97. 10.1111/jocn.16797. 37303290 10.1111/jocn.16797

[22] Dalal HJA, Ramoo V, Chong MC, Danaee M, Aljeesh YI, Rajeswaran VU. The mediating role of work satisfaction in the relationship between organizational communication satisfaction and organizational commitment of healthcare professionals: a cross-sectional study. Healthcare. 2023;11(6):806. 10.3390/healthcare11060806. 36981463 10.3390/healthcare11060806 PMC10048567

[23] Hansson M, Dencker A, Lundgren I, Carlsson I-M, Eriksson M, Hensing G. Job satisfaction in midwives and its association with organisational and psychosocial factors at work: a nation-wide, cross-sectional study. BMC Health Serv Res. 2022;22(1):436. 10.1186/s12913-022-07852-3. 35366877 10.1186/s12913-022-07852-3 PMC8976984

[24] Ericson-Lidman E, Åhlin J. Assessments of stress of conscience, perceptions of conscience, burnout, and social support before and after implementation of a participatory action-research-based intervention. Clin Nurs Res. 2017;26(2):205–23. 26671898 10.1177/1054773815618607

[25] Munkeby H, Bratberg G, Devik SA. Registered nurses’ exposure to high stress of conscience in long-term care. Nurs Ethics. 2023;30(7–8):1011–24. 10.1177/09697330231167542. 37163482 10.1177/09697330231167542 PMC10710004

[26] Gustavsson K, Fors A, van Diepen C, Axelsson M, Bertilsson M, Bångsbo A, et al. Higher levels of person-centred care are associated with lower levels of stress of conscience in hospital and municipal care: cross-sectional findings from the PCC@Work project. BMC Health Serv Res. 2025;25(1):937. 10.1186/s12913-025-13077-x. 40624661 10.1186/s12913-025-13077-x PMC12232739

[27] van Diepen C, Lood Q, Gustavsson K, Axelsson M, Bertilsson M, Hensing G, et al. Person-centred care and the work-related health and job satisfaction of health and social care professionals: protocol for a prospective longitudinal cohort study combined with qualitative studies (the PCC@Work project). BMC Health Serv Res. 2024;24(1):683. 10.1186/s12913-024-11148-z. 38816736 10.1186/s12913-024-11148-z PMC11138012

[28] Von Elm E, Altman DG, Egger M, Pocock SJ, Gøtzsche PC, Vandenbroucke JP. The strengthening the reporting of observational studies in epidemiology (STROBE) statement: guidelines for reporting observational studies. Int J Surg. 2014;12(12):1495–9. 10.1016/j.ijsu.2014.07.013. 25046131 10.1016/j.ijsu.2014.07.013

[29] Janlöv N, Blume S, Glenngård AH, Hanspers K, Anell A, Merkur S. Sweden: Health system review. (No. 25(4). Health Systems in Transition. 2023: i–198. https://iris.who.int/bitstream/handle/10665/372708/9789289059473-eng.pdf?sequence=8. 38230685

[30] Edvardsson D, Fetherstonhaugh D, Nay R, Gibson S. Development and initial testing of the person-centered care assessment tool (P-CAT). Int Psychogeriatr. 2010;22(1):101–8. 10.1017/S1041610209990688. 19631005 10.1017/S1041610209990688

[31] Åhlin J, Ericson-Lidman E, Norberg A, Strandberg G. Revalidation of the perceptions of conscience questionnaire (PCQ) and the stress of conscience questionnaire (SCQ). Nurs Ethics. 2012;19(2):220–32. 10.1177/0969733011419241. 22354810 10.1177/0969733011419241

[32] Berthelsen H, Westerlund H, Bergström G, Burr H. Validation of the Copenhagen psychosocial questionnaire version III and establishment of benchmarks for psychosocial risk management in Sweden. Int J Environ Res Public Health. 2020;17(9):1–22. 10.3390/ijerph17093179. 10.3390/ijerph17093179 PMC7246423 32370228

[33] IBM Corporation. IBM SPSS Statistics for Windows (Version 29.0) [Computer software]; 2022.

[34] Hayes AF. Introduction to mediation, moderation, and conditional process analysis: a regression-based approach. 2nd edition. The Guildford press; 2018.

[35] Faul F, Erdfelder E, Lang A-G, Buchner A. G*power 3: a flexible statistical power analysis program for the social, behavioral, and biomedical sciences. Behav Res Methods. 2007;39(2):175–91. 10.3758/BF03193146. 17695343 10.3758/bf03193146

[36] Bell ML, Fairclough DL, Fiero MH, Butow PN. Handling missing items in the Hospital Anxiety and Depression Scale (HADS): a simulation study. BMC Res Notes. 2016;9(1):479. 10.1186/s13104-016-2284-z. 27770833 10.1186/s13104-016-2284-z PMC5075158

[37] Pallant J. SPSS Survival Manual: a step by step guide to data analysis using IBM SPSS (7th ed.). Routledge; 2020. 10.4324/9781003117452.

[38] Clement LM, Bradley-Garcia M. A step-by-step tutorial for performing a moderated mediation analysis using PROCESS. Quant Methods Psychol. 2022;18(3):258–71. 10.20982/tqmp.18.3.p258.

[39] Gustavsson K, van Diepen C, Fors A, Axelsson M, Bertilsson M, Hensing G. Healthcare professionals’ experiences of job satisfaction when providing person-centred care: a systematic review of qualitative studies. BMJ Open. 2023;13(6):e071178. 10.1136/bmjopen-2022-071178. 37295826 10.1136/bmjopen-2022-071178 PMC10277035

[40] Zhang J, Rehman S, Addas A, Ahmad J. Influence of work-life balance on mental health among nurses: the mediating role of psychological capital and job satisfaction. Psychol Res Behav Manag. 2024;17:4249–62. 10.2147/PRBM.S497305. 39679316 10.2147/PRBM.S497305 PMC11646404

[41] Dziurka M, Jedynak A, Jurek K, Dobrowolska B. Correlation of nurses’ and midwives’ stress of conscience with hospital ethical climate and moral sensitivity. Sci Rep. 2025;15(1):17276. 10.1038/s41598-025-02180-y. 40389535 10.1038/s41598-025-02180-y PMC12089517

[42] Glasberg A-L, Eriksson S, Norberg A. Burnout and ‘stress of conscience’ among healthcare personnel. J Adv Nurs. 2007;57(4):392–403. 10.1111/j.1365-2648.2007.04111.x. 17291203 10.1111/j.1365-2648.2007.04111.x

[43] Ahmed A, van den Muijsenbergh METC, Vrijhoef HJM. Person-centred care in primary care: what works for whom, how and in what circumstances? Health Soc Care Community. 2022;30(6):e3328–41. 10.1111/hsc.13913. 35862510 10.1111/hsc.13913 PMC10083933

[44] Forsgren E, Feldthusen C, Wallström S, Björkman I, Bergholtz J, Friberg F, Öhlén J. Learning from the implementation of person-centred care: a meta-synthesis of research related to the Gothenburg framework. Front Health Serv. 2025;5.10.3389/frhs.2025.1589502. 10.3389/frhs.2025.1589502 PMC12259557 40666166

[45] Grover S, Fitzpatrick A, Azim FT, Ariza-Vega P, Bellwood P, Burns J, et al. Defining and implementing patient-centered care: an umbrella review. Patient Educ Couns. 2022;105(7):1679–88. 10.1016/j.pec.2021.11.004. 34848112 10.1016/j.pec.2021.11.004

